# Bacillus coagulans (PROBACI) in treating constipation-dominant functional bowel disorders

**DOI:** 10.1097/MD.0000000000020098

**Published:** 2020-05-08

**Authors:** Chen-Wang Chang, Ming-Jen Chen, Shou-Chuan Shih, Ching-Wei Chang, Jen-Shiu Chiang Chiau, Hung-Chang Lee, Yang-Sheng Lin, Wei-Chen Lin, Horng-Yuan Wang

**Affiliations:** a Division of Gastroenterology, Department of Internal Medicine, MacKay Memorial Hospital, Taipei Campus; b MacKay Junior College of Medicine, Nursing and Management; c MacKay Medical College, New Taipei City; d Institute of Traditional Medicine, National Yang-Ming University, Taipei; e Department of Medical Research, MacKay Memorial Hospital, Taipei Campus; f Division of Gastroenterology and Nutrition, Department of Pediatrics, MacKay Memorial Hospital, Hsinchu Campus; g Department of Pediatrics, Taipei Medical University, Taipei, Taiwan.

**Keywords:** *Bacillus coagulans*, firmicutes/bacteroidetes ratio, functional bowel disorders, microbiota, probiotics

## Abstract

*Bacillus coagulans* (PROBACI) bacteria have been examined for efficacy against infectious or inflammatory bowel diseases. The aim of this observational and cross-sectional study was to evaluate the effects of PROBACI against various functional bowel symptoms.

Thirty-eight enrolled patients (36.5 ± 12.6 years) with functional bowel disorders in a gastrointestinal clinic were administered PROBACI (300-mg formulation containing 1 × 10^9^ colony-forming units of *B coagulans*) twice/day over a 4-week period. Abdominal pain, abdominal distention, and global assessment were evaluated using a 5-point visual analog scale. The defecation characteristics, discomfort level, and effort required for defecation were recorded. The gut-microbiota composition in terms of the Firmicutes/Bacteroidetes ratio was analyzed by 16S-ribosomal RNA gene sequencing with stool samples at days 0, 14, and 28 post-treatment.

The 38 patients achieved significant improvements in abdominal pain (2.8 ± 0.5 to 3.3 ± 0.7, *P* = .0009), abdominal distention (2.5 ± 0.7 to 3.2 ± 0.8, *P* = .0002), and global assessment (2.7 ± 0.6 to 3.6 ± 0.7, *P* = .0001) from days 0 to 14. Compared with the diarrhea group, the constipation group achieved greater improvements in terms of discomfort during defecation (2.5 ± 0.7 to 3.1 ± 0.7, *P* = .02) and normalization of defecation style (50% vs 7.1%, *P* = .007) by day 28. A difference was observed in the Firmicutes/Bacteroidetes ratio between the constipation-dominant group (118.0) and diarrhea-dominant group (319.2), but this difference was not significant.

PROBACI provided control of abdominal pain, less discomfort during defecation, and a more normalized defecation style, especially in the constipation-dominant group.

## 1 Introduction

The use of probiotics to enhance gut health has been proposed for many years. When probiotics are consumed in sufficient quantities, they offer health benefits in preventing and treating a diverse spectrum of gastrointestinal (GI) disorders, such as antibiotic-associated diarrhea (including *Clostridium difficile*-associated), small bowel bacterial overgrowth, infectious diarrhea (including traveller's diarrhea and viral diarrhea), inflammatory bowel disease, and irritable bowel syndrome (IBS).^[1]^ Many mechanisms have been identified whereby probiotics enhance gut health, including competition for limited nutrients, inhibiting the epithelial invasion of pathogens, and augmenting the growth of nonpathogenic bacteria.^[2]^ Probiotics can decrease immune-mediated activation, modulate epithelial immune functions,^[3,4]^ and modify neural traffic between the gut and the central nervous system to alter gas transit and visceral hypersensitivity.^[5,6]^

### 1.1 Functional bowel disorder

The term “functional bowel disorder” (FBD) refers to various gastrointestinal symptoms, such as abdominal pain/discomfort, bloating/distension, and diarrhea/constipation, for which there is no obvious organic cause. FBD can lead to impaired social function and a diminished quality of life. The precise pathophysiology of FBD remains unknown. FBD continues to pose a therapeutic challenge in that the currently available therapies only provide symptomatic relief, but do not alter the natural history of the disorder. Antispasmodics may improve FBD symptoms by relaxing gut smooth muscles, providing benefits for abdominal pain and global assessment.^[7]^ However, dose-dependent adverse events, including constipation, fatigue, dry mouth, dizziness, and blurred vision may occur, especially in the elderly.

### 1.2 Current status of probiotics in functional bowel disorder

Currently, the most well studied probiotics are the lactic acid bacteria, particularly *Lactobacillus* and *Bifidobacterium* spp. However, the novel spore-forming probiotic strain *Bacillus coagulans* is relatively resistant to extreme temperatures, gastric juice, and bile salts and can survive in the gut for several days without repeated oral consumption.^[8]^ These characteristics make it a relatively ideal probiotic due to its stability and long survivability when consumed. Evidence suggests that *B coagulans* can decrease the occurrence of abdominal pain and bloating in subjects with inflammatory bowel disease and can ameliorate the symptoms of IBS or FBD.^[9–11]^

Based on these findings, we undertook an observational clinical trial to evaluate the effectiveness of *B. coagulans* [PROBACI; Standard Chemical & Pharmaceutical Co., Ltd., Taiwan] in treating various functional bowel symptoms. The results of this trial will provide clinicians a rationale in selecting the best species or strains for use in treating a particular symptom.

## 2 Materials and methods

### 2.1 Study design

Thirty-eight patients (mean age 36.5 ± 12.6 years; 5 males and 33 females) presenting with FGD in the GI clinic of Mackay Memorial Hospital from June to November, 2015 were prospectively enrolled in this observational, cross-sectional study. All patients were between 20 and 70 years of age and had self-reported symptoms, including abdominal pain/cramps and abdominal distention/bloating/flatulence for at least 1 week in the last 3 months at outpatient clinics. All subjects were otherwise in good health without progressively worsening symptoms, unexplained weight loss, nocturnal diarrhea, rectal bleeding, melena, and unexplained iron-deficiency anemia. They were willing and able to comply with the protocol and, if female, were neither pregnant nor lactating and were willing to use a reliable method of birth control. Exclusion criteria for entering this study included experiencing a cerebrovascular accident, Parkinson's disease, a history of GI cancer, previous stomach or intestinal surgery, and inflammatory bowel disease.

The participants were instructed to begin taking 1 capsule of PROBACI (300 mg containing 1 × 10^9^ colony-forming units (cfu) *B. coagulans*; Standard Chemical and Pharmaceutical Co., Ltd., Taiwan) twice per day (at approximately the same time each day) and to continue doing so for the duration of the study. Patients were permitted to use their previously prescribed medication, except that additional newly prescribed agents would interfere with the natural flora of the gut. After the study period, there was no standard maintenance therapy and the physicians can treat the patient according to the clinical condition. This observational trial was approved by the Institutional Review Board of MacKay Memorial Hospital (14 CT 032b).

### 2.2 Outcome measurements and assessments

Patients were seen for 3 visits over the course of 4 weeks, including a screening visit on day 0, and 2 follow-up visits on days 14 and 28. Patients were evaluated every 2 weeks over a 4-week period using validated questionnaires and biochemical testing of liver and renal functions. Abdominal pain (pain/clamping), abdominal distention (distention/bloating/flatulence), and global assessment were recorded, using a 5-point visual analog scale (with a score of 1 representing the greatest likelihood of having a symptom). Compliance was measured via the pill-counting method. Adverse effects were recorded. The participants also recorded their defecation frequency, fecal characteristics, and the discomfort and efforts required for defecation throughout the examination period.

The patients were divided to constipation- or diarrhea-dominant subgroups, based on their responses to the questionnaires on day 0. The constipation-dominant subgroup was recognized as having hard or lumpy stools, a defecation frequency of <3 times per week, and difficultly in defecation, whereas the diarrhea-dominant subgroup was recognized as having loose or watery stools, a defecation frequency of >3 times per day, or urgent defecations. All patients signed the IRB-approved informed-consent.

### 2.3 Gut-microbiota composition

The gut-microbiota composition in terms of the Firmicutes/Bacteroidetes (F/B) ratio was analyzed by sequencing 16S ribosomal RNA genes from stool samples on days 0, 14, and 28. Briefly, whole stools were collected in sterile boxes and immediately stored at −20 for further analysis. Stool samples were used for DNA extraction with the E.Z.N.A. Stool DNA Kit (Omega Bio-Tek, Norcross, GA), according to manufacturer's instructions. The final elution volume was 100 μL, and the DNA concentration was determined using a NanoDrop 2000 Spectrophotometer (Thermo Scientific). Amplification and detection of DNA by real-time quantitative polymerase chain reaction (Q-PCR) experiments was performed using an ABI 7500 Sequence Detection System apparatus, with system software version 1.2.3 (Applied Biosystems). Duplicate DNA samples were routinely used for Q-PCR analysis, and the mean values were calculated. Q-PCR reactions were performed in a total volume of 20 μL. Bacteroidetes and Firmicutes were detected using the Maxima SYBR Green/ROX qPCR Master Mix (Thermo Scientific), with 100 nM each of the forward and reverse primers and 1 ng DNA used for each reaction. The PCR conditions used for DNA amplification were 50°C for 2 min, 95°C for 10 min, and 40 cycles of 95°C for 15 s and 60°C for 1 min. Melting-curve analysis was performed after amplification. The primer pairs for all target regions within the 16S rRNA gene of various groups of bacteria were selected to represent important bacterial groups in the gut environment.^[12]^ The 2 primer pairs targeting the Firmicutes and Bacteroidetes 16S rRNA genes were chosen to assess and compare the relative abundances of these predominant phyla of the microbiota. Two primer pairs targeted different regions of the 16S rRNA genes of stool microbiota, and the relative F/B gene ratio was calculated.

### 2.4 Statistical analysis

In this study, considering type I error = 0.05, study power = 0.8, and expecting one score difference between before and after treatment in abdominal pain in a five-point Likert scale (expected mean differences 0.4, standard deviation 0.8), and also considering at least 10% drop out rate, the study sample size was calculated as 38 subjects.

The χ^2^ test was used to analyze categorical data. Because of the ordinal and categorical nature of the data, the Mann–Whitney *U* test was also applied to compare the data. Continuous variables were expressed as the mean ± standard deviation and compared using Student's *t* test. All statistical tests were 2-tailed, statistical significance was defined as *P* < .05, and all data analysis was performed using SAS software, version 9.2.

## 3 Results

### 3.1 Overall outcomes and adverse reactions

**Figure 1:**
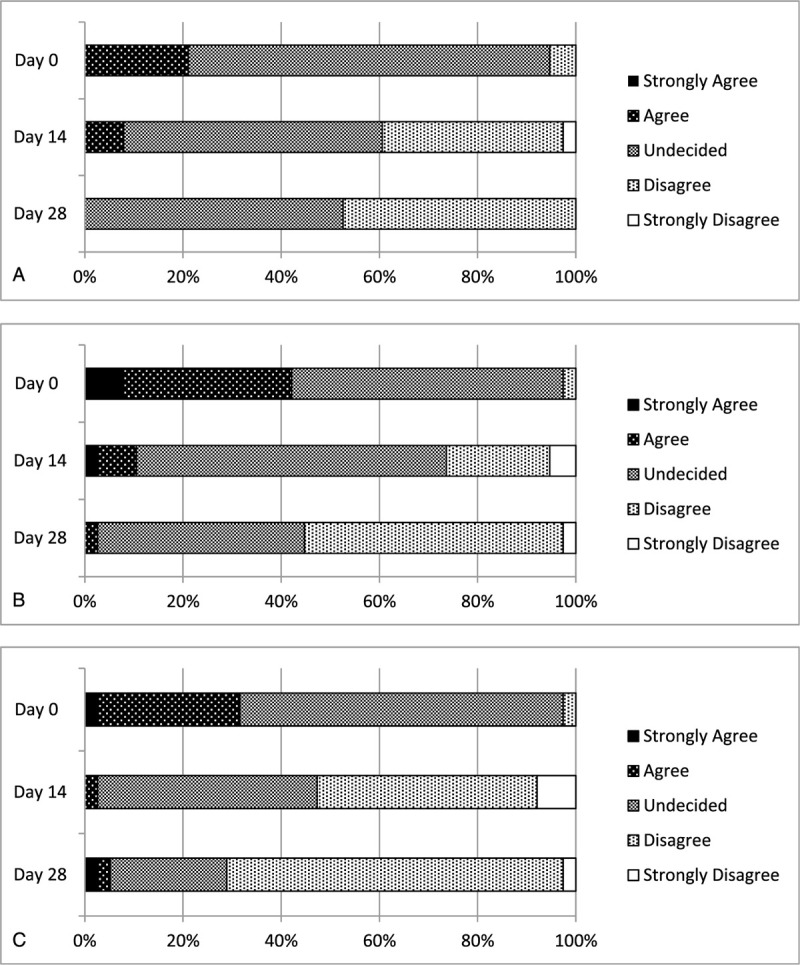
(A) Abdominal pain score of the patients after treatment from day 0, day 14, and day 28 in all patients (n = 38). Significant differences were found in week 14 (*P* = .0009) and week 28 (*P* < .00001) compared with week 0 (analysis with Mann–Whitney *U* test). (B) Abdominal distention score of the patients after treatment from day 0, day 14, and day 28 in all patients (n = 38). Significant difference were found in week 14 (*P* = .0002) and week 28 (*P* < .00001) compared with week 0 (analysis with Mann–Whitney *U* test). (C) Global assessment score of the patients after treatment from day 0, day 14, and day 28 in all patients (n = 38). Significant difference were found in week 14 (*P* < .00001) and week 28 (*P* < .00001) compared with week 0 (analysis with Mann–Whitney *U* test).

Thirty-eight patients were enrolled (age 36.5 ± 12.6 years; 5 males and 32 females; Table 1). Overall, the 38 patients achieved significant improvements comparing to baseline in abdominal pain (2.8 ± 0.5 to 3.3 ± 0.7, *P* = .0009), abdominal distention (2.5 ± 0.7 to 3.2 ± 0.8, *P* = .0002), and global assessment (2.7 ± 0.6 to 3.6 ± 0.7, *P* = .0001) from days 0 to 14, with a score of 1 representing the greatest likelihood of having a symptom (Fig. 1A–C). These improvements were maintained for abdominal pain (3.3 ± 0.7 to 3.5 ± 0.5), abdominal distention (3.2 ± 0.8 to 3.6 ± 0.6), and global assessment (3.6 ± 0.7 to 3.7 ± 0.7) from days 14 to 28 (Fig. 1A–C).

**Table 1:**
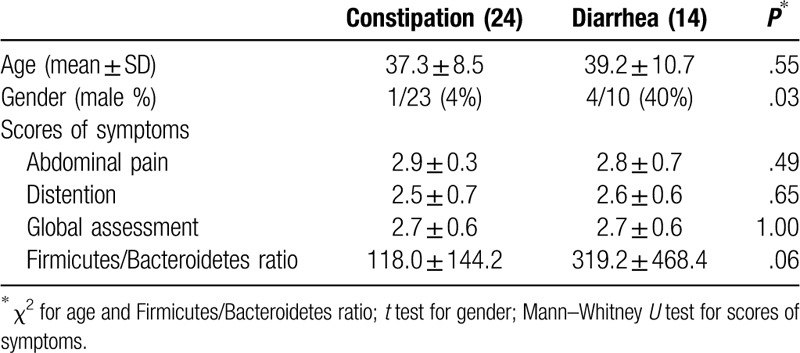
Baseline and descriptive characteristics in the constipation and diarrhea dominant groups.

None of the enrolled patients were taking concomitant medications during the study, and drug compliance was higher than 95% among all patients during the study period. No significant interactions or adverse reactions were identified during this study.

### 3.2 Subgroup analysis according to the constipation- and diarrhea-dominant groups

Twenty-four patients were recognized as constipation-dominant and 14 patients were diarrhea-dominant, according to their responses to the questionnaires on day 0. In the constipation-dominant group, patients achieved significant improvements in abdominal pain (2.9 ± 0.3 to 3.4 ± 0.7, *P* = .0009), abdominal distention (2.5 ± 0.7 to 3.1 ± 0.7, *P* = .0019), and global assessment (2.7 ± 0.6 to 3.5 ± 0.6, *P* < .0001) from days 0 to 14. These improvements were maintained in terms of abdominal pain (3.4 ± 0.7 to 3.4 ± 0.5), abdominal distention (3.1 ± 0.7 to 3.5 ± 0.6), and global assessment (3.5 ± 0.6 to 3.6 ± 0.8) from days 14 to 28 (Table 2). In the diarrhea-dominant group, patients achieved significant improvements in abdominal distention (2.6 ± 0.7 to 3.4 ± 1.0, *P* = .034) and global assessment (2.7 ± 0.6 to 3.7 ± 0.8, *P* = .002) from day 0 to day 14. The diarrhea group did not achieve significant improvements in abdominal pain (2.8 ± 0.7 to 3.2 ± 0.7, *P* = .12) from day 0 to day 14, but achieved significant improvements (2.8 ± 0.7 to 3.6 ± 0.5, *P* = .0025) by day 28. These improvements were maintained for abdominal distention (3.4 ± 1.0 to 3.6 ± 0.6) and global assessment (3.7 ± 0.8 to 3.8 ± 0.6) from days 14 to 28 (Table 2).

**Table 2:**
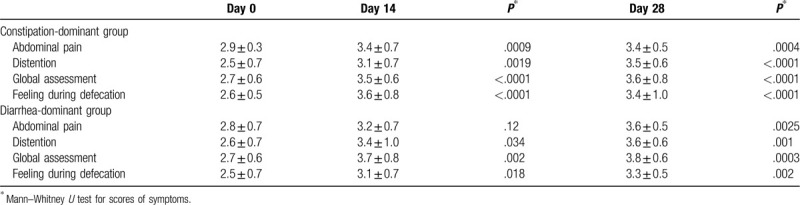
Subgroup analysis according to the constipation- and diarrhea-dominant groups.

### 3.3 Feelings after defecation and changes in defecation styles

In the constipation-dominant group, patients achieved significant improvements in their feelings during defecation (2.6 ± 0.5 to 3.6 ± 0.8, *P* < .0001) from days 0 to 14. These improvements were maintained from days 14 to 28 (3.6 ± 0.8 to 3.4 ± 1.0). In the diarrhea-dominant group, patients achieved significant improvements in their feelings during defecation (2.5 ± 0.7 to 3.1 ± 0.7, *P* = .018) from days 0 to 14. These improvements were maintained from days 14 to 28 (3.1 ± 0.7 to 3.3 ± 0.5) (Table 2). At day 14, the defecation styles improved in 37.5% of the members of the constipation-dominant group, but only in 7.1% of the diarrhea-dominant group (*P* = .04). At day 28, the defecation styles had improved in 50% of the constipation-dominant group, but still had improved on 7.1% of the diarrhea-dominant group (*P* = .007). The effects of PROBACI on changes of defecation styles were better in patients with constipation (Table 3).

**Table 3:**
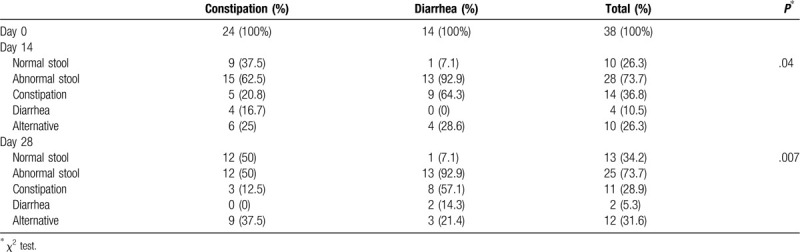
Change of defecation styles in the constipation and diarrhea dominant groups.

### 3.4 The F/B ratio

**Figure 2:**
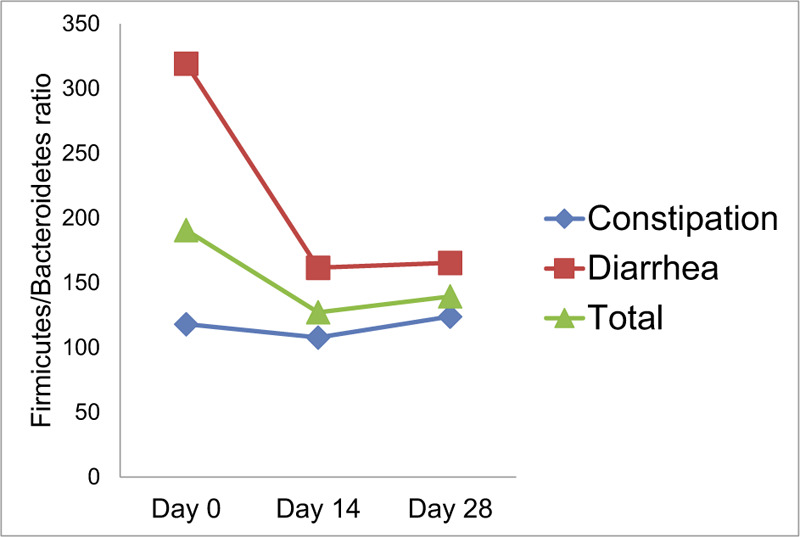
The change of the Firmicutes/Bacteroidetes ratio during the treatment period.

In terms of the F/B ratio at day 0, we observed a difference between the constipation- (118.0 ± 144.2) and diarrhea-dominant groups (319.2 ± 468.4), although statistical significance was not reached due to the small sample size (*P* = .06). We observed that the F/B ratio evolved during the treatment period, decreasing from 319.2 to 165.3 in the diarrhea-dominant group and increasing from 118.0 to 123.8 in the constipation-dominant group. The F/B ratio reached a median level between both groups of 139 (Fig. 2).

## 4 Discussion

The term “FBD” describes a problem associated with how well the stomach and bowels function. IBS and functional dyspepsia represent a spectrum of FBDs. FBD features various GI symptoms, such as abdominal pain/discomfort, diarrhea/constipation, and/or bloating/distension, for which there are no obvious underlying causes. The precise pathophysiology of FBD remains unknown. Some research efforts have focused on 2 principal targets, namely dysmotility and altered visceral sensation. It has been suggested that some patients with FBD may have a bacterial overgrowth or imbalance.^[13,14]^ The increasing understanding of gut flora–mucosa interactions and results from basic research collectively support a role for inflammatory and immune processes in the enteric neuromuscular dysfunctions of FBD.^[15]^

A natural question is why probiotics should be used to treat FBD? Probiotics can influence gut functions through promoting changes in the enteric flora, for example, by augmenting the predominance of lactobacilli/bifidobacteria or the elimination of pathogens, in order to counteract pathogen-related inflammation or fermentation.^[4,16]^ Inhibiting bacterial fermentation by modulating the flora composition could alleviate gas-related symptoms and gas transport.^[17,18]^ An effect on bloating may be the most consistent and cardinal effect of probiotics observed across all studies involving groups with constipation or diarrhea. Overall, the 38 patients in our study achieved significant improvements in abdominal pain, abdominal distention, and global assessment from days 0 to 14. These improvements were maintained from days 14 to 28, irrespective of their being constipation-dominant or diarrhea-dominant. How long the improvement can be maintained? In one study with adult IBS patients treated with *B coagulans* three times a day for 12 consecutive weeks, which showed *B coagulans* can improve abdominal pain and diarrhea in IBS patients and maintain up to 12 months.^[10]^ However, due to our study is a 4 weeks observational study only, we did not had enough evidence to answer the improvement or maintenance of symptoms after stopping the treatment.

What are the effects of probiotics on the luminal contents that change defecation behaviors? Probiotics can alter the stool volume or composition, or increase intestinal mucus secretion.^[19,20]^ In turn, these effects could influence the intestinal handling capacity to modulate constipation and diarrhea patterns. The effects on gut flora and luminal contents may affect gastroesophageal reflux or modify proximal gastric relaxation, which contribute to gastro-colic reflux and successful defection.^[21,22]^

*B coagulans* have been examined for their effectiveness in treating infectious or inflammatory bowel diseases. A double-blind, placebo-controlled randomized trial was conducted with healthy volunteers in Japan, wherein *B coagulans* in soy pulp powder was used to improve bowel movements and stool characteristics.^[23]^ When the functionally constipated patients consumed *B coagulans* (Lilac LAB, 1 × 10^8^ cfu) once per day for 2 weeks, the average scores of the self-reported scores for fecal size, sensation of incomplete evacuation, and defecation frequency significantly improved, compared to that observed in the placebo group (*P* < .05). However, in non-functionally constipated patients, no significant improvements were observed, compared to the placebo group. In our study, the constipation-dominant group achieved greater improvements than did the diarrhea-dominant group in terms of the feeling during defecation (2.6–3.6 vs 2.5–3.1) and normalization of defecation style (50% vs 7.1%, *P* = .007) at day 28.

Why were the levels of improvement different between the constipation- and diarrhea-dominant groups? Whether the improvements were accompanied by quantitative or qualitative changes in the gut bacterial flora remains a contentious issue. The accurate description of this bacterial community remains a challenge, owning to limitations in culturing and isolation techniques. Thus, we used current molecular methods by sequencing 16S ribosomal RNA genes in order to obtain a more accurate description of the microbiota composition. In healthy adults, 80% of the identified fecal microbiota can be classified into 3 dominant phyla: Bacteroidetes, Firmicutes, and Actinobacteria.^[24]^ In general terms, the F/B ratio is regarded to be of significant relevance in the human gut-microbiota composition.^[25]^ Mariat et al observed that the F/B ratio evolves during different life stages in humans.^[24]^ Previously evidence suggested that an increase of Firmicutes and a decrease of Bacteroidetes contributed to obesity and impaired regulation of fat metabolism, whereas a decreased F/B ratio has been directly related to weight loss.^[26]^ Until now, it was not possible to assess how the fecal microbiota F/B ratio predicts clinical responses. A decrease F/B ratio after 14 days of rifaximin treatment of non-constipated IBS patients was noted, however, although no differences in fecal microbiota between treatment responders vs. nonresponders was observed.^[27]^

Fecal microbial ecology was altered in cancer with the evidence of F/B ratio significantly increased in patients developing diarrhea (relative to that observed in patients that did not develop diarrhea), prior to radiotherapy to pelvic cancer.^[28]^ Based on the F/B ratios observed in our study, a difference occurred between the constipation-dominant group (118.0) and diarrhea-dominant group (319.2). These differences failed to reach statistical significance (*P* = .06), which may due to the relatively small sample size involved. We observed that the F/B ratio evolved during the treatment period, with the ratio in both groups reach a more intermediate level between the 2 groups (median of 139). Probiotics may play a role in balancing the F/B ratio. However, many unresolved issues remain that can be answered only by conducting well-designed clinical trials.

It will be important to define more clearly the mechanisms of action of various probiotics. This effort will provide a scientific rationale for physician in selecting the best species or strains against a particular symptom. This study has several limitations, including the small number of patients enrolled and gender bias in the study, which prevented a more powerful statistical analysis of the F/B ratio. In addition, this study was designed in treating various functional bowel symptoms according to clinical scenario, but not only for chronic constipation or diarrhea. Further study is needed for the population of chronic constipation to validate the efficacy. The treatment duration at least 4 weeks was in accordance with the Rome Committee's recommendation for designing treatment trials.^[29]^ Furthermore, as recommended by the committee, at least a 6-month follow-up period is required to establish the long-term efficacy. Although our study yielded promising results, further trials with longer follow-ups are warranted.

## 5 Conclusion

PROBACI achieved faster control of abdominal pain, more satisfactory feelings of defecation, and a greater proportion of patients reporting a normalized defecation style, especially in the constipation-dominant patients.

## Acknowledgments

The authors want to thank Miss Pei-Chia Liu and Mr. Yao-His Chang for preparing the documents and providing technical assistance.

## Author contributions

All authors read and approved the final manuscript.

**Conceptualization:** Horng-Yuan Wang.

**Data curation:** Chen-Wang Chang, Ming-Jen Chen, Ching-Wei Chang, Jen-Shiu Chiang Chiau, Yang-Sheng Lin, Hung-Chang Lee, Wei-Chen Lin, Horng-Yuan Wang.

**Formal analysis:** Chen-Wang Chang.

**Methodology:** Shou-Chuan Shih.

**Writing – original draft:** Chen-Wang Chang.

**Writing – review & editing:** Ming-Jen Chen.
